# Cost-effectiveness of tuberculosis infection prevention and control interventions in South African clinics: a model-based economic evaluation informed by complexity science methods

**DOI:** 10.1136/bmjgh-2022-010306

**Published:** 2023-02-15

**Authors:** Fiammetta Maria Bozzani, Nicky McCreesh, Karin Diaconu, Indira Govender, Richard G White, Karina Kielmann, Alison D Grant, Anna Vassall

**Affiliations:** 1 Department of Global Health and Development, London School of Hygiene & Tropical Medicine, London, UK; 2 TB Centre, London School of Hygiene & Tropical Medicine, London, UK; 3 Institute of Global Health and Development, Queen Margaret University Edinburgh, Musselburgh, UK; 4 Africa Health Research Institute, School of Laboratory Medicine and Medical Sciences, College of Health Sciences, University of KwaZulu-Natal, Durban, Kwa-Zulu Natal, South Africa; 5 Department of Public Health, Institute of Tropical Medicine, Antwerp, Belgium

**Keywords:** Health economics, Tuberculosis, Control strategies

## Abstract

**Introduction:**

Nosocomial *Mycobacterium tuberculosis* (*Mtb*) transmission substantially impacts health workers, patients and communities. Guidelines for tuberculosis infection prevention and control (TB IPC) exist but implementation in many settings remains suboptimal. Evidence is needed on cost-effective investments to prevent *Mtb* transmission that are feasible in routine clinic environments.

**Methods:**

A set of TB IPC interventions was codesigned with local stakeholders using system dynamics modelling techniques that addressed both core activities and enabling actions to support implementation. An economic evaluation of these interventions was conducted at two clinics in KwaZulu-Natal, employing agent-based models of *Mtb* transmission within the clinics and in their catchment populations. Intervention costs included the costs of the enablers (eg, strengthened supervision, community sensitisation) identified by stakeholders to ensure uptake and adherence.

**Results:**

All intervention scenarios modelled, inclusive of the relevant enablers, cost less than US$200 per disability-adjusted life-year (DALY) averted and were very cost-effective in comparison to South Africa’s opportunity cost-based threshold (US$3200 per DALY averted). Two interventions, building modifications to improve ventilation and maximising use of the existing Central Chronic Medicines Dispensing and Distribution system to reduce the number of clinic attendees, were found to be cost saving over the 10-year model time horizon. Incremental cost-effectiveness ratios were sensitive to assumptions on baseline clinic ventilation rates, the prevalence of infectious TB in clinic attendees and future HIV incidence but remained highly cost-effective under all uncertainty analysis scenarios.

**Conclusion:**

TB IPC interventions in clinics, including the enabling actions to ensure their feasibility, afford very good value for money and should be prioritised for implementation within the South African health system.

**WHAT IS ALREADY KNOWN ON THIS TOPIC**

- *Mycobacterium tuberculosis* transmission in clinics is a public health concern in South Africa.
- Guidelines for nosocomial infection prevention and control (IPC) are available but implementation is limited: barriers to successful implementation at both strategic and operational level are documented in the literature.
- Complexity science methods that consider the necessary enablers to overcome these barriers can inform economic evaluations to assist priority setting around investments in IPC.

**WHAT THIS STUDY ADDS**

- Interventions codesigned with tuberculosis (TB) IPC stakeholders in South Africa to include health system strengthening enablers are highly cost-effective.
- Two interventions were cost saving compared with base case: optimising the use of the existing Central Chronic Medicines Dispensing and Distribution system; and building modifications to improve ventilation.
- Operational-level enablers, including improved training and supervision and community sensitisation activities, are relatively inexpensive and do not affect the cost-effectiveness of IPC interventions.

**HOW THIS STUDY MIGHT AFFECT RESEARCH, PRACTICE OR POLICY**

- Investments in TB IPC, including additional budget to enable implementation, should be prioritised in South Africa, and similar settings.
- Low-cost enablers to ensure feasibility of TB IPC interventions in clinic settings can be identified by local and national stakeholders.

## Introduction

Primary care clinics are important sites of *Mycobacterium tuberculosis* (*Mtb*) transmission, as evidenced by the higher incidence of latent *Mtb* infection among healthcare workers compared with the general population in high burden countries.^1^ A recent modelling analysis concluded that, in a high HIV setting, transmission in these facilities may contribute up to 10.7% of disease overall,^2^ partly driven by the fact that both people with infectious tuberculosis (TB) and people with increased susceptibility to disease progression are more likely to spend time in clinics.

Guidelines for nosocomial TB infection prevention and control (IPC) are widely available.^3 4^ However, systematic reviews have identified several constraints to their successful implementation ranging from operational barriers such as lack of training, inadequate physical space and supplies of protective equipment, poor risk perception and stigma around TB, to more strategic level factors such as weak organisational culture, scarcity of funding and ineffective occupational health policies.^5–7^ Recognising the importance of the wider health system drivers of *Mtb* transmission in clinics, the Umoya omuhle (‘good air’ in isiZulu) project adopted a multidisciplinary ‘whole systems’ approach to understand this complexity and to design IPC interventions that are appropriate and feasible to scale up to an optimal level of coverage in the South African context.^8^

TB is a major public health concern in South Africa, which experienced rates of incidence and mortality among the highest in the world in 2020, driven by HIV.^9^ Umoya omuhle sought to assess the cost-effectiveness of the TB IPC interventions codesigned by stakeholders in South Africa using system dynamics modelling (SDM) techniques. SDM is a complexity science method, originating in operations research in industrial processes, that is increasingly applied in health policy and systems research,^10 11^ including in a recent analysis of the health system determinants of TB mortality in South Africa.^12^ Its focus on health systems as complex adaptive systems, which allows for the translation of this complexity in intervention design,^13^ makes it ideally suited to guide the selection of feasible, appropriate and scalable IPC interventions in a given context. Using stakeholder elicitation, SDM reveals feedback loops in the system and the non-linear effects that may result in unintended outcomes from the introduction of new interventions. SDM, therefore, can be applied to defining health system strengthening investments, or ‘enablers’, that support TB IPC fidelity, feasibility and sustainability.^14 15^ This granular understanding of dynamic system behaviour may also be used to inform agent-based models, which estimate intervention impact; and these model outputs, in turn, can be linked with resource requirement estimates to assess the value for money of health system investments.^16^

The aim of this study is to estimate the value for money of TB IPC investment in the South African health system, including enabling interventions. To our knowledge, Umoya omuhle is the first study applying SDM in conjunction with an agent-based model to inform priority setting for TB IPC. Thus, we also aim to demonstrate this novel research method that can be used to assess cost-effectiveness of complex interventions more broadly.

## Methods

We conducted a cost-effectiveness analysis of TB IPC interventions compared with a counterfactual scenario without interventions (base case) for the general population in KwaZulu-Natal. Health benefits of the interventions were measured in terms of the disability-adjusted life-years (DALYs) averted. We conducted the analysis from the perspective of healthcare providers over a 10-year time horizon.

### Patient and public involvement

A reflexivity statement covering all components of our international research partnership is included in the online supplemental file. Patient advocates and health practitioners were among the stakeholders involved in selecting the TB IPC interventions modelled in this study. Stakeholders involved in the participatory approach described below were then invited to take part in monthly virtual meetings to refine model assumptions and, finally, in a series of virtual results dissemination events (these were not held in person in South Africa, as originally planned, due to the COVID-19 pandemic).

10.1136/bmjgh-2022-010306.supp1 Supplementary data

### Intervention scenarios

The TB IPC interventions to be evaluated were selected using group model building, a participatory SDM technique used for the qualitative elaboration of causal loop diagrams.^17^ The group model building methods used in the Umoya omuhle study are described in detail elsewhere.^18^ Briefly, two 1-day workshops were held in KwaZulu-Natal in August 2019. Participants were purposively selected to capture a wide range of insights into the complex problem of TB IPC implementation in South African clinics. We started by mapping all categories of stakeholders relevant to the topic and sourcing contact details, then the list was expanded using snowball sampling. The final sample of participants included national-level and provincial-level policy stakeholders on the first day and practitioners including patient advocates, healthcare staff and managers, programme leadership, architecture and infrastructure specialists on the second day. During the workshops, causal loop diagrams depicting the key elements and interactions within the TB IPC system were elaborated, drawing from the participants’ experience and shared understanding of the dynamics at play in shaping *Mtb* transmission at clinic level, as well as from qualitative data from the Umoya omuhle study. The variable elicitation and causal loop diagram elaboration activities were designed based on published group model building scripts.^19 20^ Participants were then asked to identify points of fragility within the system where intervention would be required and, focusing on these areas, to free-list interventions that would address transmission in primary care clinics. Lastly, the interventions were ranked based on their perceived feasibility and impact, and their underlying pathways of action were refined during monthly check-in calls with participants.

The intervention scenarios included are described in table 1. Briefly, we modelled: (1) improving ventilation by regularly opening windows and doors; (2) building modifications (retrofits); (3) installation of overhead ultraviolet germicidal irradiation (UVGI) lamps in clinic spaces; (4) surgical mask wearing for patients and N95 respirators for staff; (5) optimising the use of the existing Central Chronic Medicines Dispensing and Distribution (CCMDD) system, which we modelled as an increase in the number of stable HIV patients collecting their medications from external pick-up points to reduce the number of clinic visits; (6) introduction of a queue management system and (7) introduction of an appointment system. Two combination scenarios were modelled: (A) interventions 3, 4, 5 and 7; and (B) interventions 3, 4, 5 and 6. The choice of interventions to include in the combination scenarios was guided by the relative feasibility ranking produced during group model building.

**Table 1:** Interventions and enablers description and annual costs at the two study facilities, 2019 US$

Intervention	Description	Impact modelling			Core activities costed	Annual intervention activity costs		Enablers costed	Annual enabler costs	
		Implementation	Reduction in transmission in clinics	Reduction in community-wide incident TB 2021–203		Clinic 1	Clinic 2		Clinic 1	Clinic 2
**1:** Improving ventilation by opening doors and windows	Clinical staff members are assigned to check that clinic windows and doors are open throughout the day	Assume that all doors and windows in the clinic are kept open at all times	55% (IQR 25%–72%)	5.3% (range 1.3%–12.5%)	One clinical staff member doing a round of the clinic every hour	14 526	5810	1-day training for all clinical staff every 3 years and intensified supervision from district. Electric heaters/fans to ensure thermal comfort.	2645	1603
**2:** Building retrofits	Modifications to building structure to improve air flow	An undefined package of retrofits sufficient to increase air changes per hour to a minimum of 2 in all rooms	45% (IQR 16%–64%)	4.3% (0.8%–11.2%)	Raising roof of waiting area, installing turbine ventilators and lattice brickwork	1232	412	None	0	0
**3:** UVGI	Overhead ultraviolet germicidal irradiation lamps in clinic spaces	Assume that appropriate and well-maintained UVGI systems are installed in all areas	77% (IQR 64%–85%)	7.4% (3.2–14.7%)	UV lights installation, maintenance, calibration and electricity	8232	4025	One-day training for all clinical staff every 3 years	3544	1103
**4:** Surgical mask wearing for patients and N95 respirators for staff	Surgical masks are provided for clinic attendees and fitted N95 respirators for clinical staff	Assume that 70% of all clinic attendees wear surgical masks 90% of the time, and clinic staff wear N95 masks 50% of the time	47% (IQR 42%–50%)	4.5% (2.1%–8.8%)	One N95 respirator per staff member every five shifts, fitted annually (50% coverage). One surgical mask per patient per visit (70% coverage)	Staff: 13 675 Patients: 5682	Staff: 4103 Patients: 2841	One-day training for all clinical staff every 3 years. Free leaflet for one in ten patients disseminated around clinic	3820	1241
**5:** Maximising use of existing CCMDD facilities	Drug pick-up points for chronic patients that are external to clinics (eg, private pharmacies)	Assume that virally supressed HIV patients (~92% of patients on ART) can have their ART appointments reduced to once every 6 months, with the rest needing monthly appointments	22% (IQR 12%–32%)	3.4% (0.7%–8.7%)	None	–	–	Half-day training for staff involved in implementation every 3 years. Once-off community workshops.	3201	1981
**6:** Queue management system	Patients are triaged and directed to wait outside in an open space until their name is called	Assume that only 15–30 patients are allowed to wait inside the clinic at a time, with the rest waiting in a large, covered, well ventilated outside waiting area	83% (IQR 76%–88%)	8.0% (3.8%–15.2%)	One nurse triaging patients and one lay staff member directing queues	3008	1504	Half-day training for staff involved in implementation every 3 years and intensified supervision from district. Once-off community workshops. Covered outdoor waiting area.	3269	2048
**7:** Appointments system	Appointment slots are assigned throughout the day for different patient groups and longer waiting times if no appointment	Assume that all adult chronic patients and a proportion of adult acute patients can given appointments, with their appointment times spaced throughout the day	62% (IQR 45%–75%)	5.9% (2.2%–12.9%)	1 hour per day for clerk to preretrieve files and record appointments. One hour for public awareness messaging in waiting area	4426	2951	Half-day training for staff involved in implementation every 3 years. Once-off community workshops.	6401	5139

ART, antiretroviral therapy; CMDD, Central Chronic Medicines Dispensing and Distribution; M&E, monitoring and evaluation; TB, tuberculosis; UVGI, ultraviolet germicidal irradiation.

### Model structure and parameterisation

Two agent-based models were built as part of the Umoya omuhle project to generate estimates of the impact of TB IPC interventions to reduce the risk of *Mtb* transmission among clinic attendees. The first model simulated the movement of attendees within the clinic space during their visits, while the second model simulated social contact behaviour in homes, clinics and other congregate settings to estimate the proportion of community TB incidence that resulted from clinic-acquired infections. The clinic-based model was parametrised using movement data collected on a single day at each of six primary care clinics in KwaZulu-Natal and five clinics in Western Cape. The community-based model was parametrised using data from a social contact survey conducted in the catchment areas of two clinics in KwaZulu-Natal. Methods, parameters and results of the modelling analyses are described briefly in table 1 and are published in full elsewhere.^21 22^ Details of how risk reduction among clinic staff was estimated are given in online supplemental file. The effects of intervention combinations were estimated using linear interpolation, based on the observed relationship between the proportion of transmission in clinic prevented by each intervention and DALYs averted (online supplemental figure S1).

**Figure 1:**
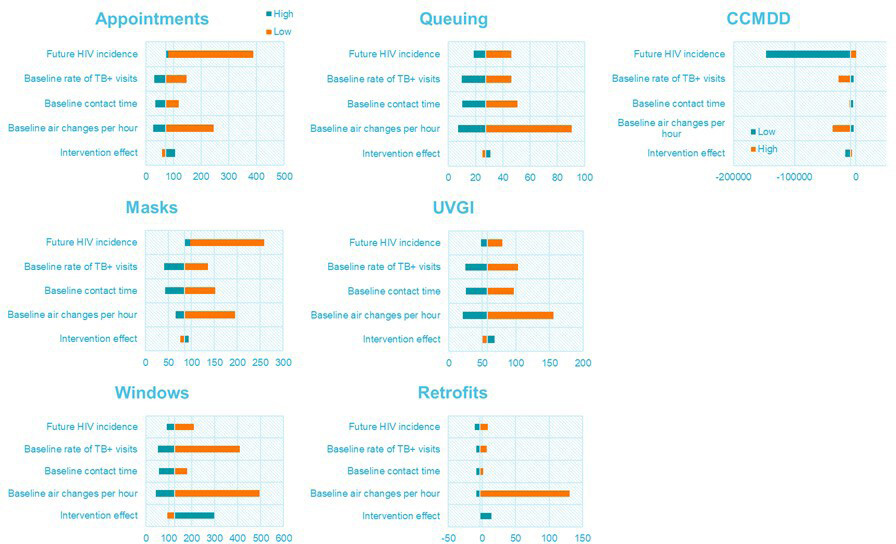
Impact of selected variables on incremental cost-effectiveness ratios, 2019 US$. CCMDD, Central Chronic Medicines Dispensing and Distribution; UVGI, ultraviolet germicidal irradiation.

### Cost data

Annual costs of core intervention activities and enablers were estimated from a provider perspective by combining price and quantity data from the published literature and local supplier quotes. A full description of the interventions and enablers cost model, alongside an explanation of how SDM results were used to inform the costing exercise, is published elsewhere.^23^ Interventions were designed and costed based on the characteristics of the two clinics in the area where the social contacts survey was conducted. Clinic 1 is a large periurban facility, while clinic 2 is a smaller rural facility in a relatively less affluent area. The intervention scenarios modelled and their annual costs at the two study clinics are presented in table 1. Capital investments and other start-up costs were annualised using a 3% discount rate for future costs.

The unit costs of consequential TB and HIV care were also derived from the published literature (table 2). All costs are presented in 2019 US$.

**Table 2:** Unit costs of consequential TB and HIV care, 2019 US$

Activities	Description	Unit	Unit cost	Source
ART cost	Drugs and clinic visits	Per patient month	60.22	MATCH study^37^
TB testing	Sum of costs of first- and second-line diagnostic tests, including visits and antibiotics	Per test	51.08	Unpublished data from XTEND trial, as calculated by Bozzani et al.^35^
TB diagnosis	One clinic visit to collect results	Per person diagnosed	5.77	Unpublished data from XTEND trial
DS-TB treatment	Facility-based observation. 2 months intensive phase, 4 months continuation phase	Per patient month	23.33	Drug prices from the Stop TB Partnership’s Global Drug Facility.^38^ Facility visit costs from Sinanovic *et al* (2015),^39^ assuming only 20% of patients are treated under DOTS, while remaining patients collect drugs from clinic once a month
MDR-TB treatment	6 months intensive phase, 12 months continuation phase	Per patient month	456.98	As for DS-TB treatment, assuming 40% of patients are hospitalised during intensive phase, the rest receive fully decentralised treatment.^39^
Short-course MDR-TB treatment	5 months intensive phase, 5 months continuation phase	Per patient month	411.94	As for DS-TB treatment. 70% of newly diagnosed MDR-TB patients assumed to be eligible for short-course treatment

ART, antiretroviral therapy; DOTS, directly observed treatment, short-course; DS-TB, drug-susceptible TB; MDR-TB, multidrug-resistant TB; TB, tuberculosis.

### Analysis

Agent-based simulations of each intervention were run for the 10-year period between 2021 and 2031. Consequential TB and antiretroviral therapy (ART) unit costs were attached to simulation outputs to estimate total diagnosis and treatment costs under different intervention scenarios. These were added to total intervention and enabler costs to calculate the incremental costs of each intervention scenario over the study period.

DALYs averted in the population were also calculated from agent-based model outputs, including deaths by age and year and the annual population distribution across TB-related and HIV-related health states. Disability weights were derived from the 2019 Global Burden of Disease study,^24^ assuming that those with active TB and either asymptomatic HIV or on ART experience the same disability as those who are HIV-negative (0.333). Remaining life expectancy over the analytic time horizon was derived from South African life tables.^25^

Intervention scenarios were then ranked based on their incremental cost-effectiveness ratios (ICERs) compared with the base case. ICERs were compared with the current cost-effectiveness threshold for healthcare priority setting in South Africa, estimated at US$3200 per DALY averted.^26^

Parameter uncertainty in the mathematical model was explored by conducting univariate sensitivity analyses around baseline ventilation rates and contact time in the clinics, the prevalence of TB in clinic attendees, and future HIV incidence. These analyses were used to construct a plausible range around the estimate of overall disease resulting from clinic transmission and of intervention impact. The sensitivity of the results to a range of other factors (the proportion of TB that resulted from transmission within households, clinic visiting rates by people with untreated HIV, and movement between high and low clinic visiting groups) was also considered in the effect modelling. The impact of these additional sensitivity analyses on reductions in TB incidence was far lower, however, and so they were not considered in the economic modelling. Full details of the univariate sensitivity analyses are reported by McCreesh *et al*.^22^

A probabilistic sensitivity analysis using a Monte Carlo simulation was conducted to explore uncertainty around cost and disability weight parameters. Cost-effectiveness ratios were recalculated 10 000 times by randomly drawing parameters from appropriate probability distributions (online supplemental table 1). Cost-effectiveness is reported over the 10-year period consisting of the average incremental costs and effects estimates and a 95% CI.

## Results

All interventions and combination scenarios modelled were highly cost-effective compared with the current opportunity cost-based threshold for South Africa (tables 3 and 4). The intervention with the highest impact compared with base case was the introduction of queue management systems with outdoor waiting areas, followed by the installation of UVGI lamps (table 3). Two interventions were cost saving compared with base case: optimising the use of the existing CCMDD system; and building retrofits to improve ventilation.

**Table 3:** TB cases in the two clinics catchment areas, incremental costs in 2019 US$, incremental DALYs averted and cost-effectiveness ratios of interventions compared with base case

Intervention	TB cases	Incremental costs, 2019 US$	Incremental DALYs averted	Incremental cost per DALY averted, 2019 US$
**Base case**	1844	–	–	–
**1:** Improving ventilation by opening doors and windows	1776	211 182	1674	126
**2:** Building retrofits	1787	−3,803	1345	cost saving
**3:** UVGI	1743	132 691	2321	57
**4:** Surgical mask wearing for patients and N95 respirators for staff	1784	218 939	1446	151
**5:** Maximising use of existing CCMDD facilities	1813	−8 413 197	980	cost saving
**6:** Queue management system	1742	68 357	2480	28
**7:** Appointments system	1772	133 241	1861	72

CCMDD, Central Chronic Medicines Dispensing and Distribution; DALYs, disability-adjusted life-years; TB, tuberculosis; UVGI, ultraviolet germicidal irradiation.

**Table 4:** Incremental costs in 2019 US$, incremental DALYs averted and cost-effectiveness ratios of combination interventions compared with the most cost-saving individual intervention (CCMDD)

Intervention combination	Incremental costs, 2019 US$	Incremental DALYs averted	Incremental cost per DALY averted, 2019 US$
**1:** CCMDD+UVGI + appointments system+mask wearing	340 377	1975	172
**2 a:** CCMDD+queuing system+UVGI + mask wearing (maximum impact)	279 557	2029	138
**2b:** CCMDD+queuing system+UVGI + mask wearing (minimum impact)	281 125	1892	149

CCMDD, Central Chronic Medicines Dispensing and Distribution; DALYs, disability-adjusted life-years; UVGI, ultraviolet germicidal irradiation.

Table 4 shows the cost-effectiveness ratios of illustrative combinations of the interventions modelled, comparing their incremental costs and effects to those of CCMDD, the individual intervention with the lowest ICER. The option with the lowest ICER, which would be recommended, is a combination of CCMDD, queuing systems with outdoor waiting areas, UVGI and mask wearing for patients (surgical masks) and staff (N95 respirators).

From the univariate sensitivity analysis, the mathematical model parameters with the largest impact on intervention ICERs were baseline ventilation rates within the clinics and rates of clinic visits by individuals with infectious TB; and future HIV incidence (figure 1). ICERs from all interventions are more sensitive to these assumptions than to uncertainty around the effects of the intervention on transmission in clinics. However, the ICERs for all interventions remained cost-effective under all assumptions tested in the univariate analysis. Probabilistic sensitivity analysis results reveal that ICERs are also robust to variations in cost and disability weight parameters (online supplemental table 2 and online supplemental figure S2 and S3). CCMDD was the intervention with the highest sensitivity to parameter uncertainty, with approximately 30% of simulations resulting in the intervention no longer being cost saving and, in 77% of these simulations, expanding CCMDD usage was no longer cost-effective. ICERs for all other interventions were below the cost-effectiveness threshold in all simulations.

## Discussion

Our study found that TB IPC interventions are an extremely efficient investment for South Africa, even when the full opportunity costs of investing in the necessary enablers to ensure effective intervention implementation are included. The wearing of surgical masks by patients and N95 respirators by clinic staff, the intervention with the highest ICER in our model, costs only US$151 per DALY averted and is considerably lower than the opportunity cost threshold for South Africa (US$3200 per DALY averted).^26^ The ICERs of TB IPC interventions in our study compare very favourably to those of other TB control measures evaluated in South Africa, such as optimising TB screening among HIV-infected patients and scaling up isoniazid preventive therapy (US$732 per DALY averted),^27^ increasing the coverage of TB diagnoses with Xpert MTB/RIF to 100% (US$1121) or implementing intensified TB case-finding among clinic attendees using the full symptoms screener recommended by the WHO (US$4190).^28^

Our analysis indicates that the South African government should not face stark choices between funding new TB IPC interventions and investing in the necessary enablers to strengthen the health system and achieve the desired level of coverage and adherence to TB care and control measures. ICERs falling significantly below the cost-effectiveness threshold indicate that both interventions and enablers are affordable and should be implemented. Moreover, many of the enablers are shared by several of the interventions in the package, so costs will further decrease when combinations are implemented, and there may be positive spill over effects from strengthening administrative and clinical processes within facilities that can benefit interventions other than TB IPC. In addition to this, all the interventions we simulated would reduce airborne transmission of other infections in clinics and many would also reduce droplet transmission. Our study predates the COVID-19 pandemic, which may have caused an increase in the uptake of measures such as ensuring spaces are well ventilated and using personal protective equipment since our model was specified. Yet, if the benefits of averting other respiratory infections (including SARS-CoV-2) were taken into account, the interventions may be even more cost-effective.

The current lack of prioritisation of TB IPC may rather stem from concerns around implementation barriers that stand in the way of achieving impact.^29^ Moreover, although the enablers included in our analysis are relatively low cost, investments in strengthening the health system may not be prioritised, especially in under-resourced systems faced with a disproportionate burden of acute care, as they may not be seen as having a direct impact on health outcomes.

The importance of considering health system constraints when assessing the cost-effectiveness of new interventions is now established in the literature.^30–32^ A real-world evaluation of Xpert MTB/RIF introduction in South Africa, for example, highlighted the importance of early consideration of enabling actions to ensure impact.^33^ In HIV, the importance of enablers has long been recognised, and included in investment analyses.^34^ However, to date, few economic evaluations informing national scale-up or health technology assessment processes attempt to quantify the costs of the enablers or the impact of health systems constraints, and incorporate them in cost-effectiveness analyses.^28 35 36^ As a result, scarce resources are wasted, or interventions are underbudgeted to achieve their full impact.

To our knowledge, our study is the first to test a systematic approach for stakeholder engagement to identify the specific constraints arising in a given setting and link that to an economic evaluation. Employing SDM to design the interventions allowed us to identify constraints that act not only at the operational level but also at the strategic, above-service level and even outside the remit of the health sector. The latter include, for example, unavailability of regular public transport links to the clinics, which impairs the successful implementation of appointment systems, or substantial time delays imposed on building retrofitting projects by the complex process for obtaining permissions and mobilising resources.^18^ Our findings on the constraints to implementation arising from ineffective administrative and clinical processes, which weaken staff morale and work culture, are in line with evidence from another recent application of SDM to explore the determinants of TB mortality in South Africa.^12^ While some of the strategic level enablers to address these constraints may not be easily quantified for inclusion in the economic evaluation, this information can still be considered as additional evidence alongside the ICERs for prioritising interventions within the proposed TB IPC package.^23^

Our study has several limitations. First, estimates of intervention effects on clinic staff were derived using a more simplistic statistical model than the transmission models used for clinic attendees. However, DALYs averted in clinic staff were only 1%–2% of those averted in the general population, and so the effects of this on the overall cost-effectiveness estimates will have been minimal. Second, we did not explore all possible intervention combinations, largely due to lack of data on their joint effects. To the extent possible, SDM evidence was used to inform decisions on which interventions to include in the combination scenarios among those that could be modelled, so that the scenarios could be as informative as possible for policymakers in terms of both cost-effectiveness and feasibility. Thus, for example, building retrofits were excluded from the combinations considered due to the time delays inherent in their implementation. Finally, we assumed in the impact modelling that each intervention would be implemented in a fixed way, and/or achieve a fixed impact on behaviour (eg, working UVGI systems would be installed in all clinic rooms, and a set level of mask wearing would be achieved). If the interventions are implemented differently (eg, due to the necessary enablers not being in place), or have different effects on behaviour, then the reductions in TB incidence and DALYs averted may be higher or lower than our estimates. Future research should focus on how the complexity of relationships in the system could be explored more dynamically by using SDM to directly inform the agent-based model structure rather than just intervention design.

Despite these limitations, our study demonstrated that investments in TB IPC represent very good value for money for South Africa, and are very cost-effective even when the costs of the necessary enablers to strengthen the health system are considered.

## Data Availability

All data relevant to the study are included in the article or uploaded as online supplemental information.
