# Supplementary material for: Cost-effectiveness of tuberculosis infection prevention and control interventions in South African clinics: a model-based economic evaluation informed by complexity science methods
Source: BMJ Glob Health. 2023 Feb 15;8(2):e010306. doi: 10.1136/bmjgh-2022-010306 (PMC9933667; doi:10.1136/bmjgh-2022-010306)
Supplement: Supplementary data [file bmjgh-2022-010306supp001.pdf]

**SUPPLEMENTARY MATERIAL**

**Cost-effectiveness of tuberculosis infection prevention and control interventions in South African clinics: A model-based economic evaluation informed by complexity science methods**

Fiammetta M. Bozzani, Nicky McCreesh, Karin Diaconu, Richard G. White, Karina Kielmann, Alison D. Grant, Anna Vassall

***Interpolation of combination interventions***

The costs and impacts were estimated for two packages of combined interventions: CCMDD, UVGI, appointment systems, and mask wearing; and CCMDD, a queuing system and outdoor waiting area, UVGI, and mask wearing.

The effect of the single interventions on DALYs averted in clinic attendees and consequential diagnosis and treatment costs was estimated using a two-stage process:

1) A model of the movement of patients through clinics, ventilation rates, and transmission risk was used to estimate the relative reduction in transmission in clinics to clinic attendees, *r*, resulting from each of the interventions
2) A community transmission model, which modelled people's social contact and transmission risk in clinics and other locations, and was used to translate the estimated impacts of the interventions on transmission within clinics only into the estimated impacts on DALYs averted, *d*, and consequential costs, *c*.

With the exception of the CCMDD intervention, which had a greater impact of risk to people on ART than to other clinic attendees, the relationship between *r* and *d* and *c* was linear, allowing the maximum numbers of DALYs that could be averted by preventing all transmission in clinics, $d_m$, to be estimated using linear interpolation (Figure S1).

Estimated reductions in transmission risk in clinics to clinic attendees, *r*, resulting from each single intervention were taken from the output of model 1[1]. The majority of the interventions considered in the combination interventions act throughout independent routes, and the combined effect of two interventions on the number of DALYs averted can therefore be estimated simply as

$$d = d_m * (r_1 + (1 - r_1) * r_2) \qquad \text{Equation 1}$$

with larger numbers of interventions combined in a similar manner.

[1] McCreesh N, Karat AS, Baisley K, Diaconu K, Bozzani F, Govender I, et al. Modelling the effect of infection prevention and control measures on rate of Mycobacterium tuberculosis transmission to clinic attendees in primary health clinics in South Africa. BMJ global health. 2021;6(10):e007124.

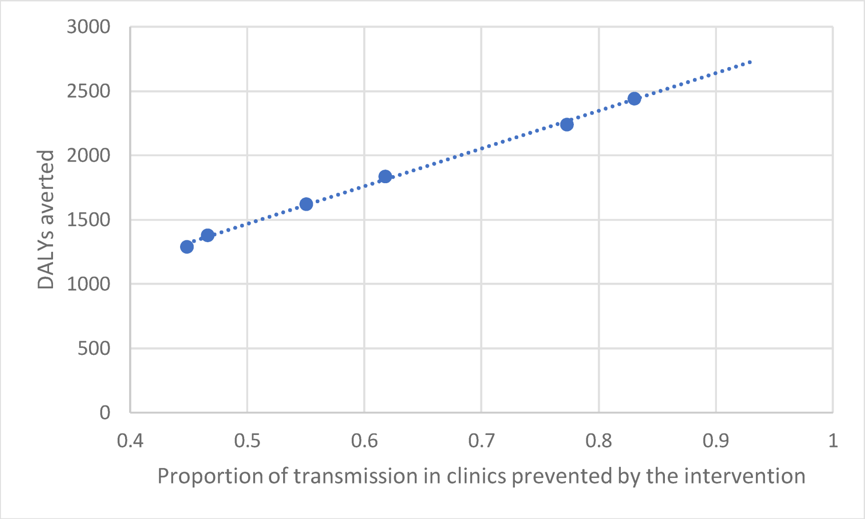


**Figure S1. Relationship between the proportion of transmission in clinics prevented by each intervention, and the number of DALYs averted.** The CCMDD intervention is not shown, as the differential impact to people on ART means that its impact on DALYs averted does not follow the linear trend of the other interventions

The exception to this was when combining the queue and UVGI interventions. The queue intervention involved having the majority of patients wait in a covered outdoor waiting area, with only the next 20 – 40 patients to be seen allowed inside the clinic to wait in the existing waiting areas. UVGI would only be installed inside the indoor waiting areas however, so there would be no effect on transmission risk to clinic attendees while they were in the outdoor waiting area. The impact of combining the queue and UVGI interventions on DALYs averted is therefore likely to be lower than would be estimated using Eq1. We therefore used Eq1 to create an upper bound on the impact of the combined interventions. To create a lower bound, we assumed that adding the UVGI intervention to an intervention package that included the queue intervention would have no additional impact on transmission to clinic attendees.

The same approach was used to estimate changes in consequential costs, and changes in DALYs averted in staff. When estimating DALYs averted in staff, the impact of combining the queue and UVGI interventions was calculated using Eq1 for both the upper and lower bound, as the majority of risk to clinic staff was assumed to occur within the clinic building.

Total costs for each intervention scenario were then calculated by adding the intervention costs to the consequential costs.

***Estimating DALYs averted in clinic staff***

The incidence of TB in clinic staff in the baseline and intervention scenarios was estimated as the incidence relative to the incidence in the general population. No data were available on excess tuberculosis risk in clinic staff in our study setting. Instead, we used estimates from a recent systematic review of TB incidence in healthcare workers, that estimated that the ratio of the rate of TB in healthcare workers compared to the general population in high burden settings was 4.32 (95% CI 2.36-7.91)[2]. In estimating relative risk to clinic staff, *r*, in the baseline scenario we sampled from the distribution uniform (2.36, 7.91). From this, we assumed that a proportion $p = 1 - (r-1)/r$ of TB in clinic staff came from transmission occurring in the general community, and that reductions in this proportion would be the same for clinic staff as for the general population. The rest of transmission to staff was assumed to occur in clinics, where the magnitude of reductions in transmission to staff was likely to be different from the magnitude of reductions to clinic attendees, due to differences in the areas where staff and attendees spent time in the clinics.

Through conversations with clinic staff, we estimated the overall proportion of time that any clinic staff spent in consultation rooms with patients vs waiting areas, and the number of staff working in the two clinics who have any indoor contact (i.e. ‘shared air’) with clinic attendees.

The rate of infection per unit time spent in waiting areas was assumed to be the same for clinic staff as for attendees, in both the baseline and intervention scenarios (with the exception of the masks intervention, where N95 mask wearing by staff was assumed to further reduce their risk). The risk of infection per working day spent in consultation rooms was estimated using the Wells-Riley equation[3], assuming:

- Seven hours of patient contact per day in consultation rooms, based on communications with clinic staff;
- A mean of 0.71-0.87 clinic attendees per room, based on observation of clinic consultation rooms;

[2] World Health Organization. WHO guidelines on tuberculosis infection prevention and control: 2019 update: World Health Organization; 2019.
[3] Riley E, Murphy G, Riley R. Airborne spread of measles in a suburban elementary school. American journal of epidemiology. 1978;107(5):421-32.

- 16% of clinic attendees are children[4].

In line with the estimates for risk in waiting areas, it was assumed that:

- 1% of adults and 0.016% of children had potentially infectious (untreated) TB[5];
- A quanta production rate of $1.25h^{-1}$ [6];
- An adult breath rate of $480lh^{-1}$ [7].

Room volumes in $m^3$ were sampled from the distribution uniform(13.7-42.8), in line with the empirical data[8]. Air changes per hour were sampled from the same distribution for consultation rooms as for waiting areas[9].

The impact of the interventions on risk to staff was estimated as follows:

- **Windows and doors.** Opening windows and doors in consultation rooms was assumed to increase ventilation rates, with the increase in air changes per hour in each consultation room sampled independently, using the same distribution as was used for waiting areas[9].
- **Retrofits.** Retrofits were assumed to increase air changes per hour (ACH) to a minimum of 12ACH in all consultation rooms. The reduction in risk to staff in waiting areas was assumed to be the same as the reduction in risk to clinic attendees.
- **UVGI.** Empirical data from studies of transmission to guinea pigs suggest that UVGI reduces the rate of transmission by 80% (95% CI 64% - 88%)[10], equivalent to a ventilation rate of 24 ACH (95% CI 9.9-62)[10]. The value of the additional quanta clearance rate was sampled from a

[4] Karat AS, McCreesh N, Baisley K, Govender I, Kallon I, Kielmann K, et al. Waiting times, occupancy density, and patient flow in South African primary health clinics: implications for infection prevention and control. MedRxiv. 2021;2021.07.21.21260806

[5] From: (1) Kunkel A, Abel Zur Wiesch P, Nathavitharana RR, Marx FM, Jenkins HE, Cohen T. Smear positivity in paediatric and adult tuberculosis: systematic review and meta-analysis. BMC Infect Dis. 2016;
(2) World Health Organization. Global tuberculosis report 2019. Geneva, Switzerland: World Health Organization; 2019; and (3) Govender I, Karat AS, Olivier S, Baisley K, Beckwith P, Dayi N, et al. Prevalence of Mycobacterium tuberculosis in sputum and reported symptoms among clinic attendees compared to a community survey in rural South Africa. Clinical Infectious Diseases. 2021.

[6] Andrews JR, Morrow C, Walensky RP, Wood R. Integrating social contact and environmental data in evaluating tuberculosis transmission in a South African township. Journal of Infectious Diseases. 2014;210(4):597-603

[7] Rudnick S, Milton DJIa. Risk of indoor airborne infection transmission estimated from carbon dioxide concentration. 2003;13(3):237-45

[8] Beckwith PG, Karat A, Govender I, Deol AK, McCreesh N, et al. Direct estimates of absolute ventilation in primary health care clinics in South Africa. MedRxiv. 2022; 10.1101/2022.03.17.22272421

[9] McCreesh N, Karat AS, Baisley K, Diaconu K, Bozzani F, Govender I, et al. Modelling the effect of infection prevention and control measures on rate of Mycobacterium tuberculosis transmission to clinic attendees in primary health clinics in South Africa. BMJ global health. 2021;6(10):e007124

[10] Mphaphlele M, Dharmadhikari AS, Jensen PA, Rudnick SN, van Reenen TH, Pagano MA, et al. Institutional tuberculosis transmission. Controlled trial of upper room ultraviolet air disinfection: A basis for new dosing guidelines. American Journal of Respiratory and Critical Care Medicine. 2015 2015/08/15;192(4):477-84

split uniform distribution with mean 24 and range 9.9 - 62%, and was implemented by additively increasing the quanta clearance rate in the Wells-Riley equation. The reduction in risk to staff in waiting areas was assumed to be the same as the reduction in risk to clinic attendees.

- **Masks.** Some data were available on the efficacy of N95 masks at reducing bacterial colonisation of respiratory infections, from a cluster randomised trial of hospital healthcare workers randomised to medical masks, N95 masks, or no intervention[11]. The study did not account for colonisation at baseline however, and could not distinguish between infections acquired at work vs in the community. In addition, some of the infections considered are acquired primarily via droplet or fomite transmission, where masks may be less effective, whereas *Mycobacterium tuberculosis* is transmitted via airborne transmission. We therefore assumed an efficacy of N95 masks of 83%, twice that found in the study. This was sampled from a split uniform distribution with mean 0.83 and range 0.72 – 0.895. We assumed that all staff who were considered to be at risk of infection from patients wore N95 masks 50% of the time. The effect on risk to staff in waiting areas was taken from the estimated reduction in risk to clinic attendees, adjusted by the estimated reductions in infection risk in clinic staff from N95 use.
- **CCMDD.** Increased coverage of CCMDD was assumed to have no effect on risk to staff in consultation rooms. The reduction in risk to staff in waiting areas was assumed to be the same as the reduction in risk to clinic attendees.
- **Queue management.** Queue management systems were assumed to have no effect on risk to staff in consultation rooms. The reduction in risk to staff in waiting areas was assumed to be the same as the reduction in risk to clinic attendees.
- **Appointment systems.** Appointment systems were assumed to have no effect on risk to staff in consultation rooms. The reduction in risk to staff in waiting areas was assumed to be the same as the reduction in risk to clinic attendees.

For each scenario, overall risk to healthcare workers in clinics was calculated as the sum of the estimated risk to each staff member in consultation rooms and waiting areas in both clinics. The relative reduction in risk in clinics, $r_c$, in each intervention scenario was then calculated as the estimated risk in the intervention scenario relative to the estimated risk in the baseline scenario.

[11] MacIntyre CR, Wang Q, Rahman B, Seale H, Ridda I, Gao Z, et al. Efficacy of face masks and respirators in preventing upper respiratory tract bacterial colonization and co-infection in hospital healthcare workers. Preventive Medicine. 2014 2014/05/01/;62:1-7.

Finally, the overall relative risk of TB (from transmission in clinics or elsewhere) in clinic staff compared to the general population in each intervention scenario was calculated as $(r - 1) * r_c + 1$.

Overall, 16,000 bootstrap estimates of the relative risk to healthcare workers were generated for each scenario. The median was 3.84 (interquartile range: 3.11-4.21) for appointment systems; 4.04 (4.32-4.56) for CCMDD; 2.05 (1.82-2.37) for masks and N95 respirators wearing; 3.16 (2.37-3.91) for queue management; 2.54 (1.81-3.43) for building retrofits; 1.63 (1.29-2.30) for UVGI and 2.57 (1.76-3.63) for windows and doors opening.

These values were used to calculate TB incidence among at-risk healthcare workers in the study clinics from community transmission model outputs. Numbers of staff with indoor patient contact were determined from discussions with clinic staff.

***Additional information on the probabilistic sensitivity analysis***

The PSA simulation was run 10,000 times, with cost parameters varied using a gamma probability distribution and disability weights varied using a beta distribution. Probability distributions used for the parameters varied in the PSA are reported in Table S1.

Scatterplots depicting incremental costs and DALYs averted over 10 years are shown in Figure S2. Average incremental costs and effects compared to base case, and 95% confidence intervals, are shown in Table S2.



Bozzani FM, *et al. BMJ Global Health* 2023; 8:e010306. doi: 10.1136/bmjgh-2022-010306

| **Variable** | **Unit** | **Distribution** | **Mean (standard deviation)** |
|---|---|---|---|
| **Unit costs** | | | |
| Appointment system | per year | Gamma (25,638.61) | 15,965 (125) |
| Queuing system | per year | Gamma (25,393.16) | 9,829 (99) |
| Surgical masks for patients | per year | Gamma (25,340.89) | 8,522 (92) |
| N95 respirators for staff | per year | Gamma (25,711.09) | 17,777 (135) |
| UVGI | per year | Gamma (25,676.08) | 16,902 (131) |
| Windows opening | per year | Gamma (25,983.35) | 24,584 (158) |
| Building retrofits | per year | Gamma (25,65.77) | 1,644 (41) |
| CCMDD | per year | Gamma (25,207.30) | 5,182 (73) |
| ART with CCMDD | per patient month | Gamma (25,24.69) | 617 (25) |
| ART | per patient month | Gamma (25,28.91) | 723 (27) |
| ART initiation | per patient month | Gamma (25,2.41) | 60 (8) |
| TB testing | per test | Gamma (25,2.04) | 51 (7) |
| TB diagnosis | per patient diagnosed | Gamma (25,0.23) | 6 (2) |
| 1st line treatment | per treatment month | Gamma (25,0.93) | 23 (2) |
| MDR treatment | per treatment month | Gamma (25,18.28) | 457 (22) |
| MDR short regimen | per treatment month | Gamma (25,16.48) | 412 (20) |
| **Disability weights** | | | |
| HIV negative, TB | | Beta (23.59,47.24) | 0.333 (0.58) |
| HIV positive, TB | | Beta (20.68,29.30) | 0.408 (0.64) |
| HIV positive, no TB | | Beta (25.58,67.77) | 0.247 (0.52) |
| ART, TB | | Beta (23.58,47.24) | 0.333 (0.58) |
| ART, no TB | | Beta (31.80,375.89) | 0.078 (0.28) |

**Table S1. Probability distributions for parameters varied in the PSA**

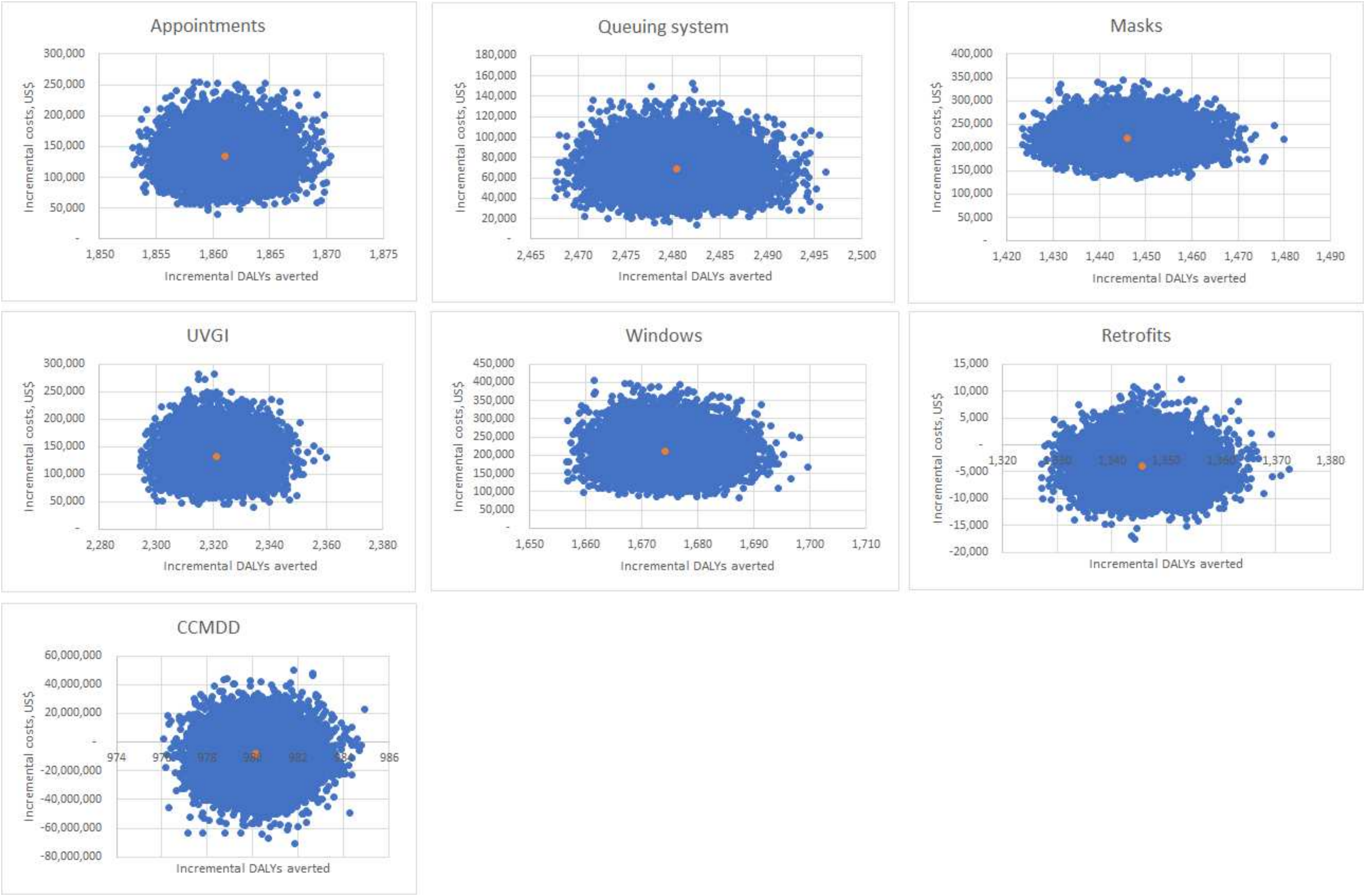


**Figure S2. Scatterplots of intervention costs and DALYs averted from PSA runs**

| Intervention | Incremental costs compared to base case | | | DALYs averted compared to base case | | | ICER, costs/DALYs |
|---|---|---|---|---|---|---|---|
| | Average | L 95% CI | U 95% CI | Average | L 95% CI | U 95% CI | |
| Appointments | 133,686 | 79,621 | 199,120 | 1,861 | 1,856 | 1,866 | 72 |
| Queuing | 68,483 | 34,475 | 108,766 | 2,480 | 2,472 | 2,489 | 28 |
| Masks | 219,403 | 167,427 | 278,640 | 1,446 | 1,446 | 1,446 | 152 |
| UVGI | 132,561 | 74,890 | 201,200 | 2,321 | 2,221 | 2,321 | 57 |
| Windows | 211,900 | 127,893 | 312,473 | 1,674 | 1,674 | 1,674 | 127 |
| Retrofits | - 3,857 | -10,510 | 3,572 | 1,345 | 1,345 | 1,345 | cost saving |
| CCMDD | - 8,307,495 | -39,383,270 | 22,068,738 | 980 | 980 | 980 | cost saving |

**Table S2. PSA results**

***Umoya Omuhle study team acknowledgments***

| **Name** | **Institution/s** |
|---|---|
| Anand Ramnanan (deceased) | AHRI |
| Anita Edwards | AHRI |
| Aruna Sevakram | AHRI |
| Dickman Gareta | AHRI |
| Duduzile Mkhwanazi | AHRI |
| Emmerencia Gumede | AHRI |
| Hannah Keal | AHRI |
| Harriet Gliddon | AHRI; UCL |
| Kobus Herbst | AHRI |
| Marlise Venter | AHRI |
| Njabulo Dayi | AHRI |
| Nkosingiphile Buthelezi | AHRI |
| Nompilo Ndlela | AHRI |
| Nompumulelo Nyawo | AHRI |
| Nonhlanhla Madlopha | AHRI |
| Nozi Khumalo | AHRI |
| Nozipho Mthethwa | AHRI |
| Nzuzo Ntombela | AHRI |
| Patrick Gabela | AHRI |
| Phumla Ngcobo | AHRI |
| Precious Zulu | AHRI |
| Raveshni Durgiah | AHRI |
| Richard Lessells | AHRI |
| Sabelo Ntuli | AHRI |
| Sanele Mthiyane | AHRI |
| Sashin Harilall | AHRI |
| Sifundesihle Malembe | AHRI |
| Sinethemba Mabuyakhulu | AHRI |
| Siphephelo Dlamini | AHRI |
| Sithembiso Luthuli | AHRI |
| Sizwe Sikhakane | AHRI |
| Sphiwe Mthethwa | AHRI |
| Tevania Naidoo | AHRI |
| Thabile Mkhize | AHRI |
| Thandekile Nene | AHRI |
| Thomas Murray | AHRI |
| Tshwaraganang Modise | AHRI |
| Vanisha Munsamy | AHRI |
| Yutu Dlamini | AHRI |
| Zilethile Khumalo | AHRI |
| Zinhle Mkhwanazi | AHRI |
| Zodwa Mkhwanazi (deceased) | AHRI |

***Author reflexivity statement***

1. **How does this study address local research and policy priorities?**

The data used in this paper is drawn from a larger project entitled Umoya Omuhle which had seen different work packages assessing different aspects of infection prevention and control for tuberculosis. Tuberculosis is a leading cause of death in South Africa. Transmission in clinics is a particular area of concern, with healthcare workers at elevated risk of the disease compared to the general population.

2. **How were local researchers involved in study design?**

The ‘Umoya Omuhle’ project was a collaborative partnership between researchers in South African and UK institutions. The South African partners have played an active and equal role throughout the project, including the design of the study in the initial grant application, and the choice and design of the infection control interventions. IG contributed to writing the initial protocol, and other South African partners contributed to drafting the fieldwork manual, protocols, and instruments for the qualitative and ethnographic data collection

3. **How has funding been used to support the local research team?**

All research activities conducted by the local research team were funded by the grant for this study.

4. **How are research staff who conducted data collection acknowledged?**

All research staff involved in data collection are acknowledged in the supplementary file.

5. **Do all members of the research partnership have access to study data?**

All members of the partnership have access to data.

6. **How was data used to develop analytical skills within the partnership?**

Research staff involved in data collection were part of concurrent data quality checking and routine reporting. Research staff either contributed by providing feedback on the analytical outputs or were part of the team doing data analysis.

7. **How have research partners collaborated in interpreting study data?**

The broader “Umoya omuhle” project had a project reference group comprised of local researchers and senior health managers who gave input on the progress of the project and early outputs.

8. **How were research partners supported to develop writing skills?**

As part of the broader project, regular writing retreats were held to encourage and support collaborative writing across the UK and South African researchers.

9. **How will research products be shared to address local needs?**

The “Umoya omuhle” project has held a number of stakeholder meetings and feedback sessions that included public health managers from all levels who were given the opportunity to engage with the research findings. In addition, a policy brief based on outputs from Umoya omuhle was written.

10. **How is the leadership, contribution and ownership of this work by LMIC researchers recognised within the authorship?**

IG is from South Africa, and is affiliated with the Africa Health Research Institute.

11. **How have early career researchers across the partnership been included within the authorship team?**

For this particular paper, we have included early career researchers (FB, NM, IG, KD) within the authorship team, including IG who is based in South Africa.

12. **How has gender balance been addressed within the authorship?**

All authors are female (FB, NM, KD, KK, ADG, AV) with the exception of RGW

13. **How has the project contributed to training of LMIC researchers?**

IG contributed to drafting the protocol and training the field team.

14. **How has the project contributed to improvements in local infrastructure?**

There was no direct or indirect impact on local infrastructure as a result of this project

15. **What safeguarding procedures were used to protect local study participants and researchers?**

Ethics approval for the study was granted by the research ethics committees of the University of KwaZulu-Natal (BE662/17) the University of Cape Town (165/2018) and the London School of Hygiene and Tropical Medicine (14872/3).
